# Preparation and Physicochemical Characterization of Hyaluronic Acid-Lysine Nanogels Containing Serratiopeptidase to Control Biofilm Formation

**DOI:** 10.1038/s41598-024-56732-9

**Published:** 2024-03-13

**Authors:** Hanieh Mahdiani, Faegheh Yazdani, Mahsa Khoramipour, Vahideh Valizadeh, Haleh Bakhshandeh, Rassoul Dinarvand

**Affiliations:** 1 Department of Pharmaceutical Nanotechnology, Faculty of Pharmacy, Tehran University of Medical Sciences, Tehran, Iran; 2 Nanotechnology Research Centre, Faculty of Pharmacy, Tehran University of Medical Sciences, Tehran, Iran; 3 Nanobiotechnology Department, New Technologies Research Group, Pasteur Institute of Iran, Tehran, Iran; 4 QC Department, Osve Pharmaceutical Co, Tehran, Iran

**Keywords:** Therapeutic enzyme, Serratiopeptidase (SRP), Hyaluronic acid (HA), Lysine, Nanogel, Bacterial biofilm, Biofilms, Drug delivery

## Abstract

Remarkable resistance of bacterial biofilms to high doses of antimicrobials and antibiotics is one of their main challenges. Encapsulation of proteolytic enzymes is one of the suggested strategies to tackle this problem. In this regard, the antibacterial and anti-biofilm activity of biocompatible hyaluronic acid- Lysine nanogels containing serratiopeptidase (SRP-loaded HA-Lys nanogel) was assessed against *P. aeruginosa* and *S. aureus* strains*.* SRP-loaded HA-Lys nanogel was prepared using dropping method and optimized by Box-Behnken experimental design. These formulations were studied for physical characterization, release profile, stability, bioactivity, and anti-biofilm effects. The particle size, polydispersity index (PDI), and surface charge were measured by *Z*etasizer Nano ZS. The average particle size and zeta potential of the optimum sample were 156 nm and -14.1 mV, respectively. SRP release showed an initial burst followed by sustained release and the highest release was around 77%. Enzyme biological activity data revealed the higher efficiency of free SRP compared to SRP-loaded HA-Lys nanogel. The time-kill assay showed that both forms of SRP-loaded HA-Lys nanogel and blank HA-Lys nanogel showed significant antimicrobial activity against examined bacteria in comparison to the free enzyme. The obtained results demonstrated improved anti-biofilm efficacy and down regulation of tested biofilm genes for both SRP-loaded HA-Lys nanogel 100% and blank HA-Lys nanogel 100% compared to SRP 100%.

## Introduction

Multicellular structures made up of densely packed, extremely hydrated populations of microorganisms embedded in a matrix of self-synthesized proteinaceous or polymeric materials are known as bacterial biofilms^1,2^. Biofilms can display biotic or abiotic connections to surfaces. They possess remarkable abilities such as strong resistance to both the innate and adaptive immune systems, along with the capacity to tolerate high amounts of antibiotics and antimicrobial agents. As a result of such traits, bacterial biofilms can cause chronic infections, introducing them as a crucial health and financial challenge^3^. It has been discovered that secreted and surface exposed proteins are essential for the development, stability, and control of biofilms^1^. Proteases were therefore proposed as a possible anti-biofilm agents, a theory that was subsequently confirmed by research. Studies using pure proteases from various organisms have shown efficacy against biofilms, with metalloproteases specifically proving to be highly influential in combating biofilm formation and growth^4^. Furthermore, the removal of biofilms has also been accomplished using commercial proteases^5^.

Serratiopeptidase (SRP), a commercially available bacterial metalloprotease, has shown effectiveness in treating a range of biofilm-associated medical disorders^6^. SRP not only can modify the virulent phenotype of bacteria in biofilms but also it is effective against mature biofilms^7^, Furtheremore, it enhances the bactericidal effect of antibiotics against biofilms^8^. SRP is a proteolytic enzyme with immense applications in therapeutic areas, which have been validated by several in vitro*, *in vivo, and clinical studies and through historical evidence. Its multifaceted properties, including anti-inflammatory, anti-biofilm, analgesic, anti-edemic, and fibrinolytic activities, are responsible for these applications^9^. However, anti-inflammatory enzymes might be preserved against proteolytic degradation and activity loss by different methods including loading them into novel delivery carriers. As a result, due to SRPs multi potential effects, various drug delivery systems were developed in the last decade, and a large number of clinical and pre-clinical studies were performed on encapsulated SRP.

Nanocarriers are currently the first choice in drug delivery systems due to their various benefits^10^. In addition, increased drug stability is one of the most important properties of nanocarriers for the delivery of proteins and polypeptides. Ionic complexes are one of the safest nanocarriers in clinical use due to their chemical reactions. It has been shown that Hyaluronic acid (HA) and l-lysine (Lys) can self-assemble to form these ionic complexes due to their negative and positive charges, respectively^11^. HA is a glycosaminoglycan (GAG) which is a natural biopolymer and extracellular matrix component composed of d-glucuronic acid and N-acetylglucosamine repeated groups^12^. The antibiofilm activity of HA has been studied in number of investigations. Drago et al. have studied the anti-adhesive and anti-biofilm activity of HA towards bacterial species commonly isolated from respiratory infections. In this study, antibiofilm activity was investigated by spectrophotometry after the biofilm was incubated with HA and stained by crystal violet^6,13^. Besides, Lys is an essential and water-soluble amino acid that was first derived from casein hydrolysis. This amino acid presents in high concentrations in organisms, and its commercial form exists as Lys monohydrochloride^14^. In this study, we showed the effectiveness of SRP loaded-HA-Lys nanogel on the biofilm removal. This work aims to create a special kind of gel using HA and Lys which contains SRP as a potent anti-biofilm agent. The present study focused on evaluating the size, stability, and release mechanism of the HA gels containing SRP, as well as their capability to maintain the enzyme activity. The anti-bacterial and anti-biofilm effects of SRP-loaded HA-Lys nanogel were assessed against *P. aeruginosa* and *S.aureus* strains. Moreover, the impact of SRP-loaded HA-Lys nanogel on the expression level of certain biofilm genes was also examined.

## Methods and materials

### Materials

SRP was purchased from Advanced Enzyme Technologies Ltd. India; HA and Lys were from Sigma-Aldrich, UK. Chloroform was obtained from Merck Company, Germany and Spectra/Por dialysis membrane (MWCO 100KDa) from Sigma-Aldrich, U.S.A. All materials and organic solvents used were of analytical grade. *S. aureus* ATCC 6538 and *P. aeruginosa* ATCC 15,442 were obtained from the microbial bank of Pasteur Institute of Iran. This study was performed in the nanotechnology department of Pasteur Institute of Iran.

### Preparation of blank HA-Lys nanogels and SRP loaded HA-Lys nanogels

SRP-loaded HA-Lys nanogels were prepared by the dropping method. In this method, the enzyme was added dropwise to the Lys solution and the resulting solution was then added to the HA solution by the dropping method. All steps were done whyle stirring. For blank HA-Lys nanogels, SRP was not added to Lys solusion.

### Optimization of SRP-loaded HA-Lys nanogel by experimental design

In order to determine the process’s effective components, this study employed the d-Optimal Design technique, and 19 trials were carried out with Design Expert software (version 7.0.10, State-Ease, Inc., Minneapolis, MN). Three factors of polymer ratio (the ratio of HA to Lys), drug concentration, and stirrer speed are selected as test variables and nanoparticle size (Z-average), polydispersity index (PDI), encapsulation efficiency (EE%), and nanoparticle charge (Zeta Potential) are considered as test responses. Range of variables were selected based on information obtained from previous studies and initial screening tests. The quantitative variables are shown in Table 1. Table 2 presents the proposed test matrices.

**Table 1:** Quantitative variables of the test and its minimum and maximum values.

Factor	Name	Unit	Low Actual	High Actual
A	Polymer ratio	w/w%	0.0125	0.0425
B	Drug concentration	g/ml	0.0075	0.0175
C	Stirrer speed	rpm	700	1100

**Table 2:** Box-Behnken design (BBD) for optimization of the blank HA-Lys nanogel.

Run	A: Polymer ratio	B: Drug concentration	C: Stirrer speed	Z-average	Zeta Potential	PDI	EE
Unit	w/w%	g/ml	rpm	nm	mV	-	%
1	0.0125	0.0075	700	266	− 11.9	0.34	86
2	0.0275	0.0125	900	201	− 11.2	0.56	82
3	0.0500	0.0125	900	182	− 10.6	0.41	79
4	0.0275	0.0125	900	209	− 13.7	0.54	95
5	0.0425	0.0175	700	161	− 11.0	0.39	92
6	0.0275	0.0125	900	189	− 11.2	0.43	89
7	0.0275	0.0200	900	152	− 11.3	0.31	90
8	0.0125	0.0175	700	151	− 30.5	0.35	91
9	0.0125	0.0075	1100	134	− 7.1	0.26	84
10	0.0275	0.0125	900	143	− 35.4	0.29	84
11	0.0425	0.0075	700	167	− 29.9	0.40	72
12	0.0275	0.0050	900	213	− 4.2	0.33	66
13	0.0500	0.0125	900	149	− 2.9	0.25	81
14	0.0425	0.0075	1100	141	− 1.6	0.27	66
15	0.0275	0.0125	900	220	− 11.8	0.43	85
16	0.0425	0.0175	1100	192	− 6.8	0.42	82
17	0.0275	0.0125	1200	205	− 3.4	0.39	80
18	0.0275	0.0125	600	234	− 8.7	0.31	82
19	0.0125	0.0175	1100	172	− 7.7	0.29	83

### Physicochemical characterization of SRP-loaded HA-Lys nanogel

#### Determination of particle size and zeta potential

*Z*etasizer Nano ZS (Malvern Instrument Ltd. Malvern, UK) was used to evaluate the mean particle size(Z-average), polydispersity index (PDI), and zeta potential of nanoparticles at room temperature (RT).

#### Determination of SRP entrapment efficiency

The centrifugation method was used to test the SRP entrapment efficiency (EE) of nanogels indirectly. Specifically, 1 ml of SRP-loaded HA-Lys nanogel solution was centrifuged at 14,000g for 45 min at 4 °C (Eppendorf 580R centrifuge, Germany). The free SRP concentration in the aqueous phase was evaluated using the Bradford method. The EE% of SRP was calculated using the following equation :

$$EE \left(\%\right)= \left[\frac{A-B}{A}\right]\times 100$$

where A is the initial SRP concentration used to prepare the nanogel and B is the un-entrapped SRP concentration measured in the supernatant.

#### Particle shape and morphology

With the use of field emission scanning electron microscopy (FE-SEM, NOVA NANOSEM 450 FEI model), the morphology of the blank HA-Lys nanogel and SRP-loaded HA-Lys nanogel was determined. The samples were coated with a layer of gold (100 Å) for 3 min under an argon atmosphere at a pressure of 0.2 atm.

### In vitro release study

The optimized SRP-loaded HA-Lys nanogel was subjected to release assay with a dialysis membrane (MWCO 100 KDa). In summary, 2 ml of the chosen formulation were placed in a dialysis tube and left to float in a constantly agitated releasing medium that contained PBS (pH 7.2) at 37 °C. Then, 1 ml of the sample was taken at 0.5, 1, 2, 4, 6, 24, 48, and 72 h, and the Bradford method was used to measure the concentration of released SRP. The same volume of fresh PBS was added to the release medium and the enzymes cumulative release was determined and plotted against the time. Ultimately, the total amount of medication discharg was calculated. Based on zero-order, first-order, Korsmeyer Peppas, and Higuchi models, the SRP release profile was produced. By using linear regression, the best-fitting model for entrapped SRP was identified.

### Fourier-transform infrared spectroscopy (FT-IR)

To analyze the interaction between SRP and excipients, FTIR spectra of SRP, HA, Lys, SRP-loaded HA-Lys nanogel, and blank HA-Lys nanogel were examined in KBr discussing a PerkinElmer FTIR spectrophotometer (spectrum Two, USA). FTIR measurements were carefully performed at ambient temperature in the scanning range of 4000 to 400 cm^-1^ at a constant resolution of 4 cm^-1^.

### Stability study

The stability of SRP-loaded HA-Lys nanogel was evaluated based on EE% and particle size measurements at 4 °C and 25 °C (RT) at different time intervals for three months.

### SRP biological activity and kinetics

The modified Lowry method was used to measure the enzymatic activity of SRP. This method identifies the phenol group in the tyrosine root and has a sensitivity of 0–2 μg/ml of protein. Non-specific measurement of protease activity may be used as a standard method to determine the activity of SRP. In this experiment, casein acts as a substrate for the protease. When the protease digests the casein, the amino acid tyrosine is released along with other amino acids and peptide fragments. Next, the Folin-Ciocalteu reagent (FCR) reacts with the released tyrosine followed by the absorbance being read at 660 nm by a spectrophotometer.A higher level of protease activity is indicated by the production of more chromophores when casein hydrolysis by the protease releases more tyrosine. The protease activity of the samples can be calculated in terms of an enzyme equivalent to tyrosine released from casein hydrolysis per minute using the tyrosine standard curve^15^.

### Time-kill assay

Time-kill test helps to understand how well antimicrobial drugs work against different types of germs. This test measures the efficacy of antimicrobial agents against various germs, depending on the quantity and duration of usage. The time-kill evaluation was done for all treatments against *S. aureus*, and *P. aeruginosa*. For this, the mixture of bacteria and treatments including SRP 100%, SRP-loaded HA-Lys nanogel 50%, blank HA-Lys nanogel 100%, and SRP-loaded HA-Lys nanogel 100% were left to sit for 2, 4, 6, 24, 48, and 72 h at 37 °C. Then, the optical density (OD_660_) was measured at particular time intervals using a microplate reader (Epoch, Japan). The bacteria growth pattern without any treatment was used as the positive control.

### In vitro antibiofilm activity (microtiter plate test)

Quantification of in vitro anti-biofilm activity of free SRP and SRP-loaded HA-Lys nanogel was performed on two strains of *S. aureus* and *P. aeruginosa*. Firstly, biofilm formation was performed by culturing *S. aureus*, and *P. aeruginosa* for 24 h in LB and Thioglycolate medium, respectively. The culture is diluted 1:100 in fresh suitable culture medium and incubated in a 96-well plate for 4–24 h at 37 °C. Biofilm formation will be assessed using a microscopic method. Then selected standard bacteria were treated with sub-MIC (1/2 MIC) values of free SRP and SRP-loaded HA-Lys nanogel, and the plates were washed with distilled water. After drying, the attached cells were stained with 1% crystal violet and washed with distilled water three times. The attached dye in the wells was solubilized with 30% v/v glacial acetic acid, and optical density (OD) was recorded at 550 nm using the ELISA reader.

### Real Time

Real-time PCR (Applied Biosystems, Carlsbard, CA, USA) was used to assess the effect of treatments on gene expression. The expression level of *ndvB* and *icaA* genes were evaluated for *P. aeruginosa* and *S. aureus*, respectively The bacteria were exposed to sub-MIC concentration of SRP 100%, SRP-loaded HA-Lys nanogel 50%, blank HA-Lys nanogel 100%, and SRP-loaded HA-Lys nanogel 100% for 24 h. Tubes containing cells were treated with a cold solution called RNX TM–PLUS. Then we mixed the tubes and left them at RT for 5 min before adding chloroform. The cells were kept on ice for 5 min and then centrifuged (4000 g, 4 °C, 15 min). The materials were transferred to RNase-free small tubes that contained the same amount of isopropanol. The mixture was centrifuged at 4 °C for 5 min. The RNA pellet was mixed with ethanol and then treated with Diethyl pyrocarbonate (DEPC) in water. RNA was removed from all parts of the cell using a special kit (Qiagen, USA). cDNA was synthesized from RNA by mixing reaction buffer, RNA, Enzyme-Mix, and water in specialized tubes. The mixture was kept in a warm place for 10 min at 25 °C and then for 60 min at 47 °C. The reaction was stopped by heating it at 85 °C for 5 min. Then, the mixture was put on ice until it was needed. We used a PCR kit from Bioneer, Korea to do PCR. 10 pmol of each primer was utilized, then the mix was heated at 95 °C for 5 min. Following that, a cycle of heating at 95 °C for 1 min, 60 °C for 1 min, and 72 °C for 30 s was repeated for 34 cycles. Finally, we heated the mix at 72 °C for 5 min. The PCR results were analyzed by comparing the CT of target genes (*ndvB*, and *icaA*), and 16S rRNA gene as control. The cells treated with nanoparticles were examined for gene activity by contrasting their mRNA levels with untreated cells. The sequence of primers for the target genes is shown in Table 3.

**Table 3:** Forward and reverse primers used in *ndvB*, and *icaA* genes detection.

Gene	Primer	Ref
IcaA	CCTAACTAACGAAAGGTAG	^42^
	AAGATATAGCGATAAGTGC	
NdvB	GGCCTGAACATCTTCTTCACC	^43^
	GATCTTGCCGACCTTGAAGAC	
16 SrRNA	AGAGTTTGATCCTGGCTCAG	^42^
	GACGGGCGGTGTGTACAA	

### Statistical analysis

Statistical analysis of d-optimal design was performed using Design-Expert 7.0.10 software (Stat-Ease Inc., U.S.A). Responses were analyzed using the ANOVA test. The *p-value* of <0.05 was considered as significant.

### Ethics approval

There are no “human subjects” in this study

## Results

### Optimizing the process of nanoparticle manufacturing

The results obtained by a BOX-Behnken experimental design investigating the interactions between three independent variables namely polymer ratio (A), drug concentration (B), and stirrer speed (C) at three levels are shown in Table 1 and 19 formulations of SRP-loaded HA-Lys nanogel were obtained as shown in Table2.

#### Effect of formulation variables on particle size

As can be seen in Table 2, SRP-loaded HA-Lys nanogel particles have a size range of 134–266 nm. Table 3 lists the results of the variance analysis of particle size. Interestingly, particle size did not significantly differ from the best-fitting model (*p-value* > 0.05)**.** Just the combination of drug concentration and stirrer speed **(B and C)** has a significant effect on particle size**.** The *p-value* for B and C was less than 0.05 (Table 4). The resulting equation in terms of coded value was as follows:

1 $${\text{Particle}}\,{\text{Size }} = { 183}.{24} - {1}.{1}*{\text{A}} - {1}0.0{4}*{\text{B}} - {11}.{93}*{\text{C}} + {15}.{26}*{\text{AB}} + {14}.{51}*{\text{AC}} + {26}.{34}*{\text{BC}}$$

**Table 4:** Analysis of variance for the quadratic polynomial model for size, PDI, EE, and zeta potential.

**Source**	**Prob > F**		**Source**	**Prob > F**	
**Size (nm)**			**EE (%)**		
Model	0.1157	Not Significant	Model	0.0052	Significant
A	0.8998		A	0.1035	
B	0.2648		B	0.0019	
B	0.1898		C	0.1715	
AB	0.1805				
AC	0.2012				
BC	0.0304				
**PDI**			**Zeta potential (mV)**		
Model	0.4459	Not Significant	Model	0.2345	Not Significant
A	0.1285		A	0.9097	
B	0.6203		B	0.62	
C	0.7620		C	0.0513	
AB	0.6950				
AC	0.8826				
BC	0.4915				

There is a converse relationship between polymer ratios, drug concentration, and stirrer speed with vesicle size. While keeping other factors constant, the increament in polymer ratio leads to a decrease in the average vesicle size. Also, there is a reduction in the vesicle size after increasing the drug concentration. Moreover, the particle size reduces with increasing the stirring time. At higher stirring speeds, formation of the smaller size emulsion droplets lead to a significant decrease in particle size.

#### Effect of formulation variables on entrapment efficiency

The entrapment efficiency of the prepared SRP-loaded HA-Lys nanogel was found to vary between 66 and 95%.The effects of independent variables on the EE are shown in Table 4. The models for entrapment efficiency were considered to be significant since their p values were less than < 0.05. Drug concentration **(B)** has a significant effect on entrapment efficiency **(***p-value* < 0.05). However, A and B have no significant effect on EE% (*p-value* > 0.05). The interaction terms of the EE are shown in the below equation:

2 $${\text{EE}} = { 82}.{58} - {2}.{8}0*{\text{A}} + {6}.0{8}*{\text{B}} - {2}.{32}*{\text{C}}$$

The data (see Table 1) revealed that drug entrapment efficiency was dependent on the polymer concentration^16^. It can be justified for ionic interaction between the drug and polymer component.

#### Effect of formulation variables on zeta potential

Zeta potential is considered as the main impact factor for product stability. Based on Table 2, the zeta potential of prepared SRP-loaded HA-Lys nanogel ranges from − 2.9 to − 35.4 mV. The analysis of variance for zeta potential is listed in Table 4. The models for zeta potential were considered to be not significant since their p values were more than > 0.05. None of the independent variables has a significant effect on zeta potential (*p-value* > 0.05). The interaction term of the zeta potential have been shown in the below equation:

3 $${\text{Zeta}}\,{\text{Potential}} = - {12}.{16} - 0.{3}*{\text{A}} - {1}.{3}*{\text{B}} + {5}.{43}*{\text{C}}$$

There is an indirect relationship between polymer ratio and drug concentration on zeta potential value. While the relationship between stirrer speed and zeta potential was direct. According to Eq. (3), zeta potential turned to be more negative while the amount of HA was increased.

#### Effect of formulation variables on polydispersity

The PDI of all nanoparticles was obtained by Box–Behnken, ranging from 0.266 to 0.558 (Table 2). The *p-values* of PDI for all independent variables in Table 4 were more than 0.05. Accordingly, the HA/Lys ratio, drug concentration, and stirring speed have no significant effect on PDI (*p-value* > 0.05). According to the regression, the polymer ratio and the amount of drug have a positive effect on PDI, while the stirring speed has a negative influence on PDI. The interaction terms of the PDI have been shown in the below equation:

$$PDI = 0.45 + 0.04*A + 0.012*B - 7.4E - 003*C + 0.012*AB + 4.5E - 003*AC + 0.021*BC - 0.041*A^{2} - 0.042*B^{2} - 0.031*C^{2}$$

#### Data optimization

Suitable nanocarriers were designed after optimization of particle size, EE%, zeta potential, and PDI. The best desirability index (desirability = 0.920) was obtained when the polymer ratio was 0.013 w/w%, the drug concentration was 0.018 g/mol, and the speed of stirring was 700 rpm (Table 5). A multi-criteria index was applied to optimize formulations. Narrow particle size, minimum polydispersity, the zeta potential of about − 30 mV, and the maximum percentage of entrapment efficacy were predicted in optimal conditions. The obtained desirability index was 0.92, which confirms the validity of optimization. The anticipated nanocarriers should have the following characteristics compromizing particle size, zeta potential, polydispersity index, and entrapment efficacy calculated as 159.147 nm, − 18.590 mV, 0.283, and 93.779, respectively. According to Table 6, the validity of the central composite design was clear because there was no significant difference between observed and predicted data for SRP-loaded HA-Lys nanogel.

**Table 5:** Determined optimal conditions.

Number	A: Polymer ratio	B: Drug concentration	C: Stirrer speed	Desirability
1	0.013	0.018	700	0.928

**Table 6:** Forecasts and obtained results under optimal conditions.

Source	Z-average (nm)	Zeta Potential (mV)	PDI	EE
Predicted	159.147	− 18.590	0.283	93.779
Observed	156.5 ± 10.9	− 14.14 ± 4.61	0.38 ± 0.07	89.66 ± 1.57
Obs/Pred	0.99	0.76	1.34	0.96

### Characterization of nanoparticles

#### Particle size and surface charge

**Figure 1:**
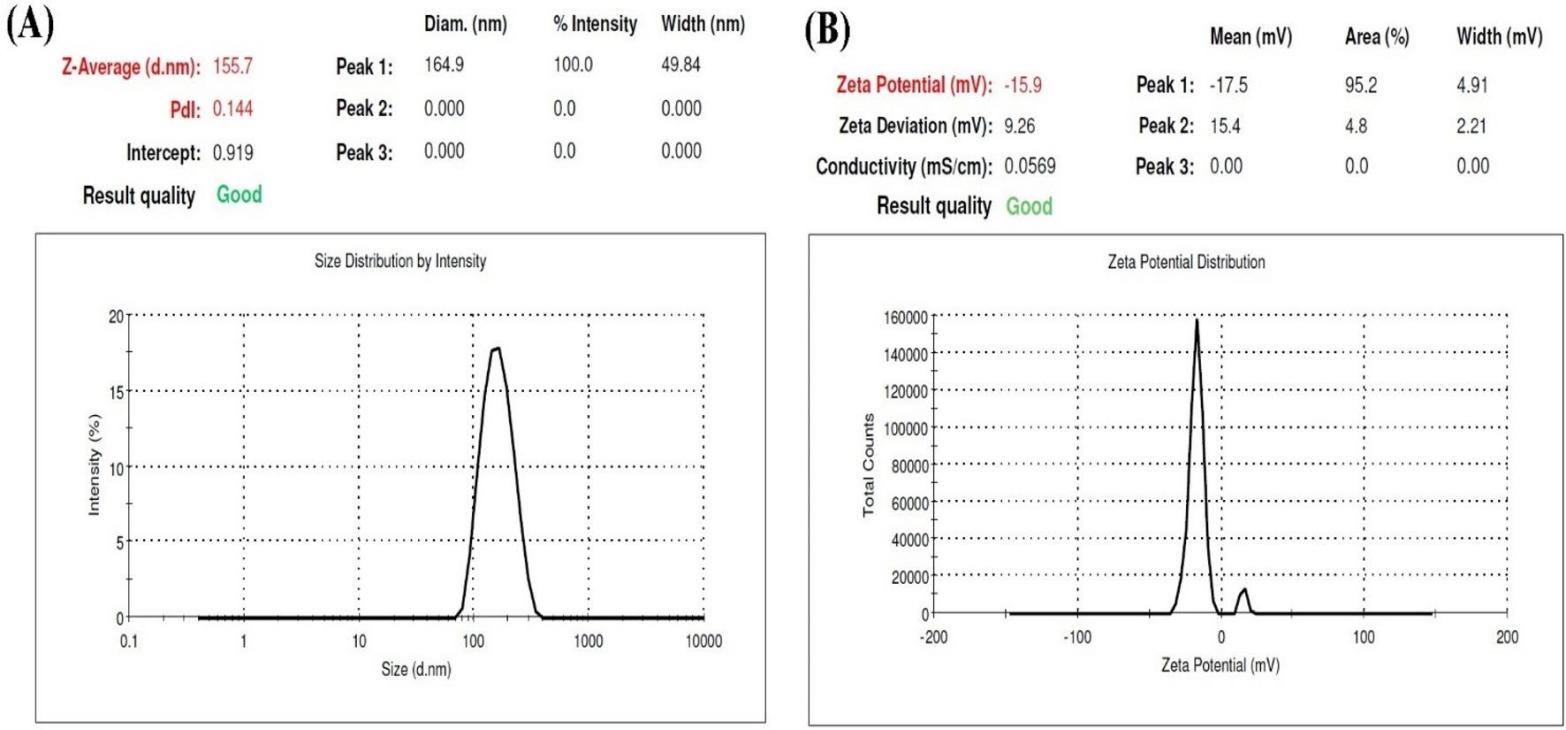
(**A**) Particle size distribution and, (**B**) Zeta potential curve of SRP-loaded HA-Lys nanogel.

The size and dispersion of the particles and their morphology were evaluated by zeta sizer. As shown in Fig. 1**,** the average particle size and zeta potential of the optimum sample were 156 nm and − 14.1 mV, respectively. A negative value of zeta potential could be as a result of the presence of carboxyl groups on the nanoparticle surfaces, indicating good stability of formulations. The stability of nanogels is affected by zeta potential, so the higher values of zeta potential results in enhanced stability as the weak electrostatic repulsive force between nanoparticles inhibits aggregation. Moreover, a narrow particle size distribution (0.144) indicates a relatively homogenous dispersion.

#### Scanning electron microscopy (SEM)

**Figure 2:**
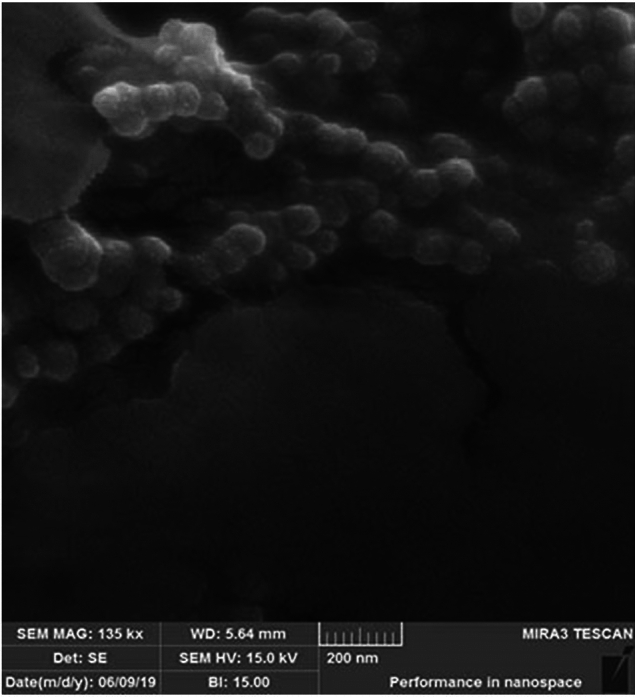
SEM images of SRP-loaded HA-Lys nanogel.

The optimized SRP-loaded HA-Lys nanogel formulation was distinctly globular with smooth surfaces, according to SEM pictures (Fig. 2). The average size of the optimized formulation is less than 100 nm.

#### Assessment of nanogel structure by FTIR

**Figure 3:**
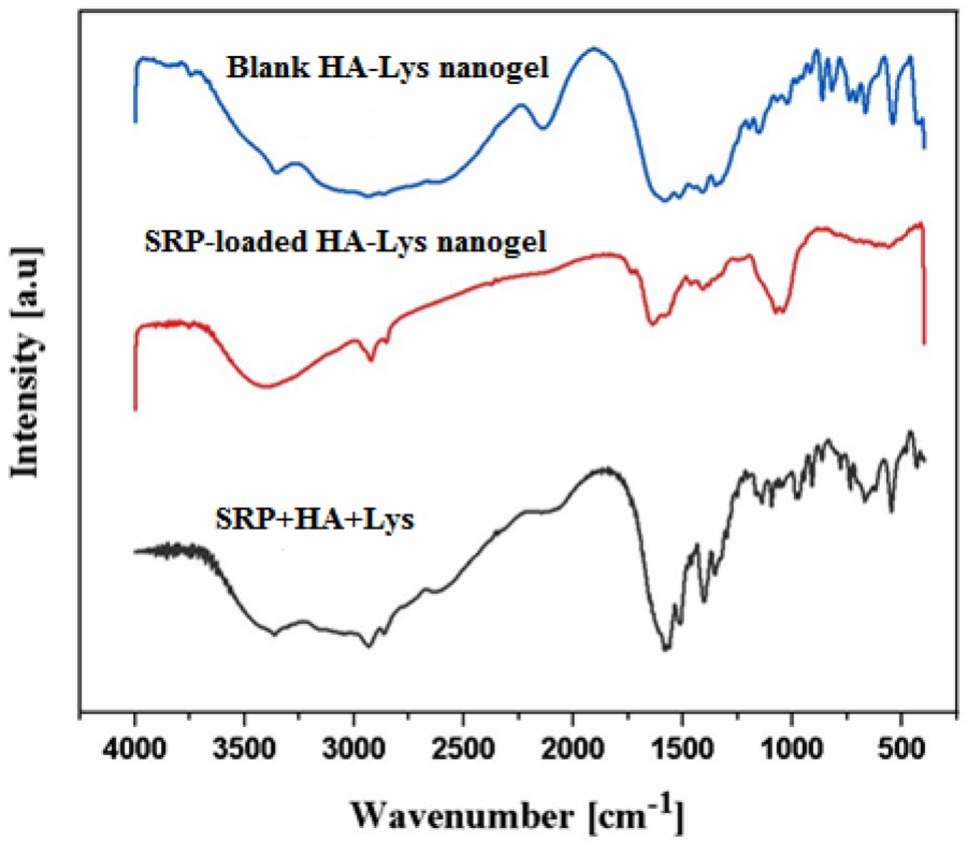
FTIR spectra of blank HA-Lys nanogel, SRP-loaded HA-Lys nanogel, a mix of the different component in the formulation.

Figure 3 shows blank HA-Lys nanogel's FTIR spectrum. Similar bands of raw minerals were displayed in the spectrum. The established HA structure included a large stretching band of hydroxyl groups, two bands corresponding to carbonyl stretching bands of carboxylic acid amide, and ether bands. Nevertheless, blank HA-Lys nanogel was not allocated the carboxylic acid of HA's carbonyl stretching band. However, Lys showed bands relating to N–H in-plane bending, C–N stretching of amine, two bands resulting from symmetric and asymmetric CH2 stretching, a carbonyl stretching band of carboxylic acid, and bands corresponding to amine groups. The quaternary ammonium salt was identified as the source of a band at 2685 cm^−1^ in the FTIR spectra of blank HA-Lys nanogel (Fig. 3). All ammonium salts exhibited a broad band between 2200 and 3300 cm^−1^, as has been covered in other articles, because of a mixture of NR4 + group bands. Thus, these findings support the ionic interaction between the amine group of Lys and the carboxyl group of HA that was postulated for self-assembled blank HA-Lys nanogel^17^. The published FTIR spectra in references were compared with the FTIR spectra of SRP in the SRP-loaded HA-Lys nanogel (Fig. 3). In light of all of the information provided, the FTIR spectrum of the SRP-loaded HA-Lys nanogels (Fig. 3) revealed no appreciable shift in the enzyme peaks following the entrapment of SRP in the nanoparticles, indicating that there is no incompatibility, either chemically or physically, between SRP and blank HA-Lys nanogels.

### Release profile

**Figure 4:**
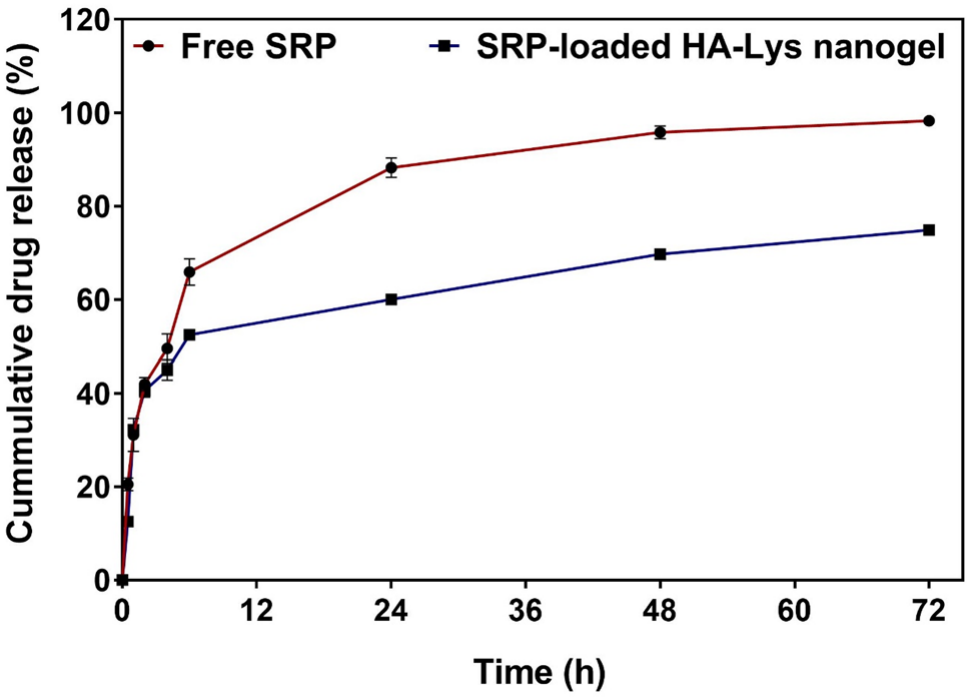
In vitro release of SRP from (**a**): SRP solution (**b**): SRP-loaded HA-Lys nanogel.

The release of SRP through the optimized structure was evaluated at physiological pH (7.4) for 72 h. As shown in Fig. 4, initially the burst release of free SRP was observed in first 8 h and were then released monotonously for the remaining 12, 20, and 24 h. The drug release of free SRP was approximately 75% during the first 8 h, and then approximately 100% of the drug was released within 48 h. However, the release of SRP from HA nanogels was about 55% after 8 h then the release was monotonous for the remaining 12, 20, and 24 h (Fig. 4). Finally, the release of the SRP reached nearly 77% after 72 h.

### Stability studies

**Figure 5:**
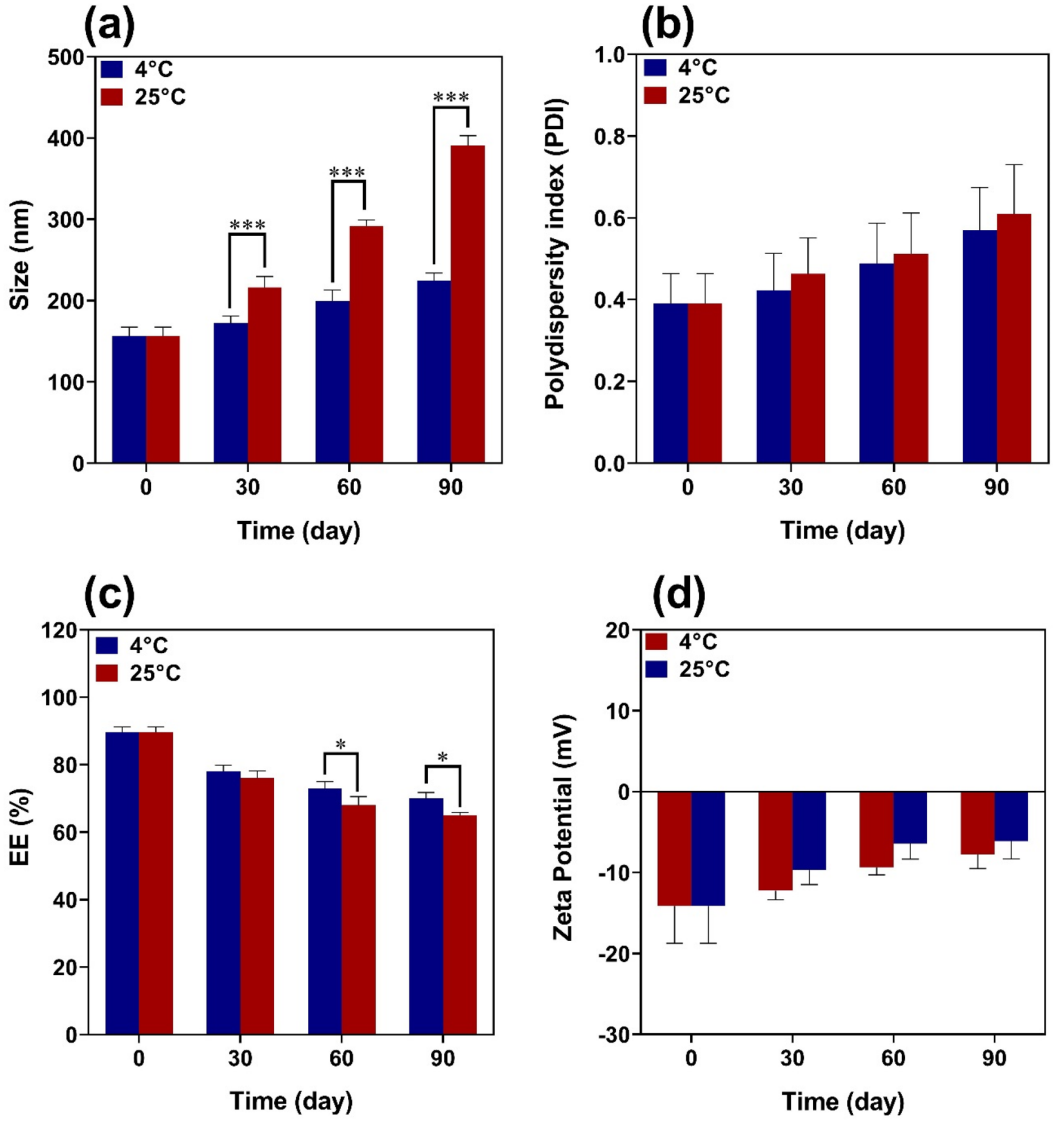
Evaluation of stability of nanoparticles at 4 and 25° C for 90 days.

The physical stability of SRP-loaded HA-Lys nanogel was investigated by characterization of vesicle size, PDI, and EE% after storage at 4 °C and 25 °C for 90 days. As can be seen in Fig. 5, an increase in the percentage of size and PDI and reduction in EE% was shown during storage time which could be due to the aggregation of nanoparticles and the leakage of the drug. Mean vesicle size, PDI, and EE% of nanogels showed fewer changes after 90 days at 4 °C in contrast to 25 °C. This can be explained by the lower stability of blank HA-Lys nanogel at higher temperatures.

### Enzyme biological activity

**Figure 6:**
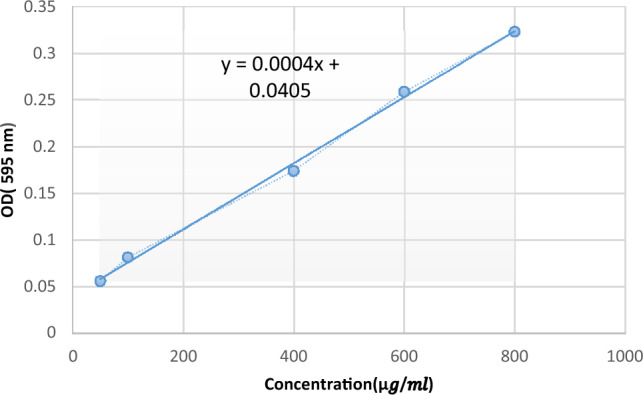
Standard curve of tyrosine.

Enzyme biological activity assay was carried out by protease activity, as described in method section, and the amount of SRP activity was calculated based on the calibration curve (Fig. 6). There was a linear relationship between the concentration of enzyme and enzyme activity. This showed that the enzyme activity assay for the determination of enzyme concentrations during formulation development was reliable and accurate. The most important factor in this study is the retention of the biological activity of the SRP after loading in blank HA-Lys nanogel. Enzymatic activity was calculated according to the following formula (Eq. 4), and the activity of free enzyme and enzyme loaded in the nanogels was compared^18^.

4 $${\text{The}}\,{\text{amount}}\,{\text{of}}\,{\text{enzyme}}\,{\text{activity}} = \left( {{\text{T}} \times {\text{Vc}}} \right) \times \left( {{\text{D}} \times {\text{Ve}}} \right)/\left( {{\text{B}} \times {\text{Vt}}} \right)$$

where T is reaction time, Vc is calorimetric volume, D is dilution factor, Ve is volume of enzyme used, B is enzyme substrate concentration and Vt is total volume

$${\text{Enzyme}}\,{\text{activity}}\,{\text{of}}\,{\text{free}}\,{\text{SRP}} = \left( {{93}0.{7} \times {2}/0.{1} \times {1}0 \times {1}} \right) \times {2} = {3722}.{8}$$

$${\text{Enzyme}}\,{\text{activity}}\,{\text{of}}\,{\text{nanogels}} = \left( {{463} \times {2}/0.{1} \times {1}0 \times {1}} \right) \times {2} = {1852}$$

The results obtained from Eq. 4 show that the activity of SRP in blank HA-Lys nanogel was less than that of the free enzyme.

### Time-kill assay

**Figure 7:**
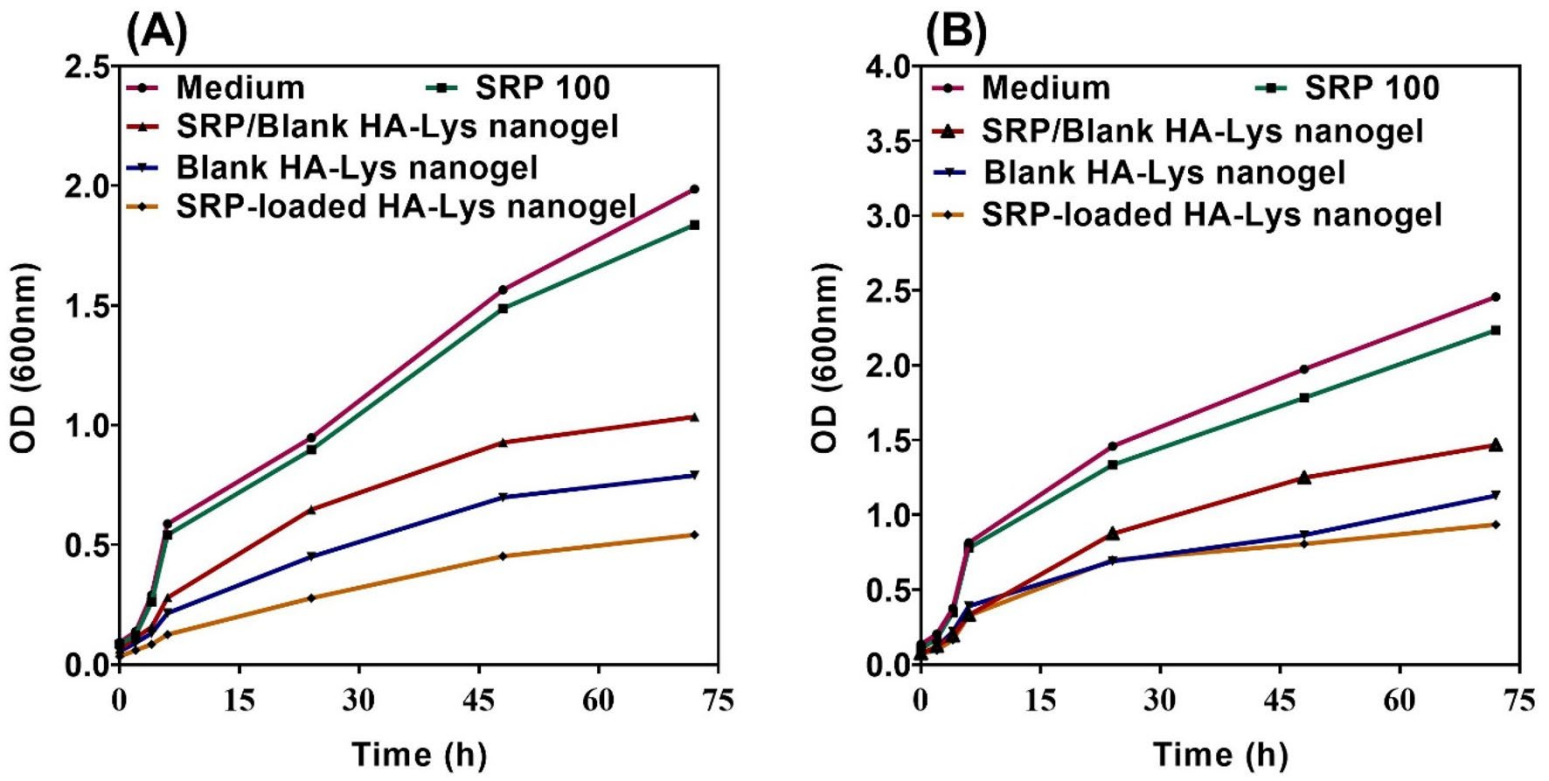
Time kills assay of (**A**) *S. aureus*, and (**B**) *P. aeruginosa.*

The time-killing assay was done using different samples to assess their effect against *P. aeruginosa* and *S. aureus* was tracked for 2 days (Fig. 7). The largest and most rapid decline against both examined bacteria was seen using SRP-loaded HA-Lys nanogel 100%.This results might be due to association of HA to the bacterial cell membrane as an antimicrobial agent, making SRP to get into the cell more efficient. According to the Fig. 7A, B, applying the SRP-loaded HA-Lys nanogel 100% leaded to the lowest growth of both examined bacteria, which means it is the most efficient on bacteria’s growth compared to other groups. Furthermore, it is evident that SRP 100% had minimal impact on the bacterial growth of tested strains.

### Antibiofilm activity assessment

**Figure 8:**
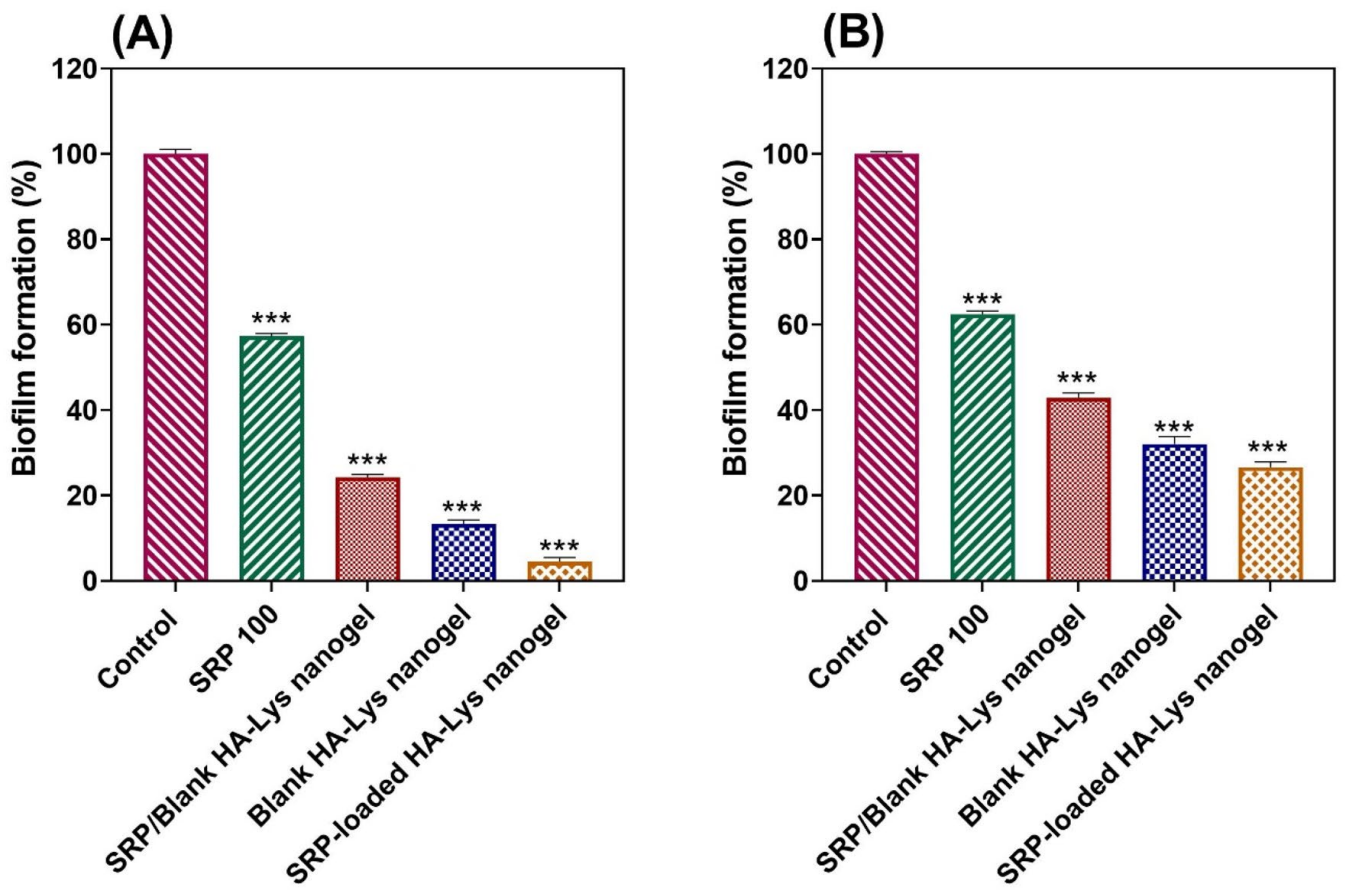
Bacterial biofilm formation rate (**A**) *S.aureus* and (**B**) *P. aeruginosa* is inversely related to the treatment effect of the substance; (Data are represented as mean ± SD; *** *p-value* < 0.001).

Figures 8A, B show the obtained results of microbiological studies. The biofilm formation rate of examined bacteria was calculated based on the following formula^19^:

$${\text{Biofilm}}\,{\text{formation}}\,{\text{rate}} = \left( {{\text{mean}}\,{\text{optical}}\,{\text{absorption}}\,{\text{of}}\,{\text{control}}/{\text{mean}}\,{\text{optical}}\,{\text{absorption}}\,{\text{of}}\,{\text{test}}} \right) \times {1}00$$

All samples showed considerable microbial activity against *P. aeruginosa* and *S. aureus* compared to the control groups (*p-value* < 0.001). The results show that the concentration of free SRP and blank HA-Lys nanogel has a converse relationship with bacterial biofilm formation. Increasing the concentration of enzymes and blank nanogels results in a decrease in the rate of biofilm of bacteria. Interestingly, the rate of biofilm formation of nanogels with SRP/blank HA-Lys nanogel 50% was about 30%, while treating bacteria with blank HA-Lys nanogel 100% was results less than 20%.

### Real-time PCR

**Figure 9:**
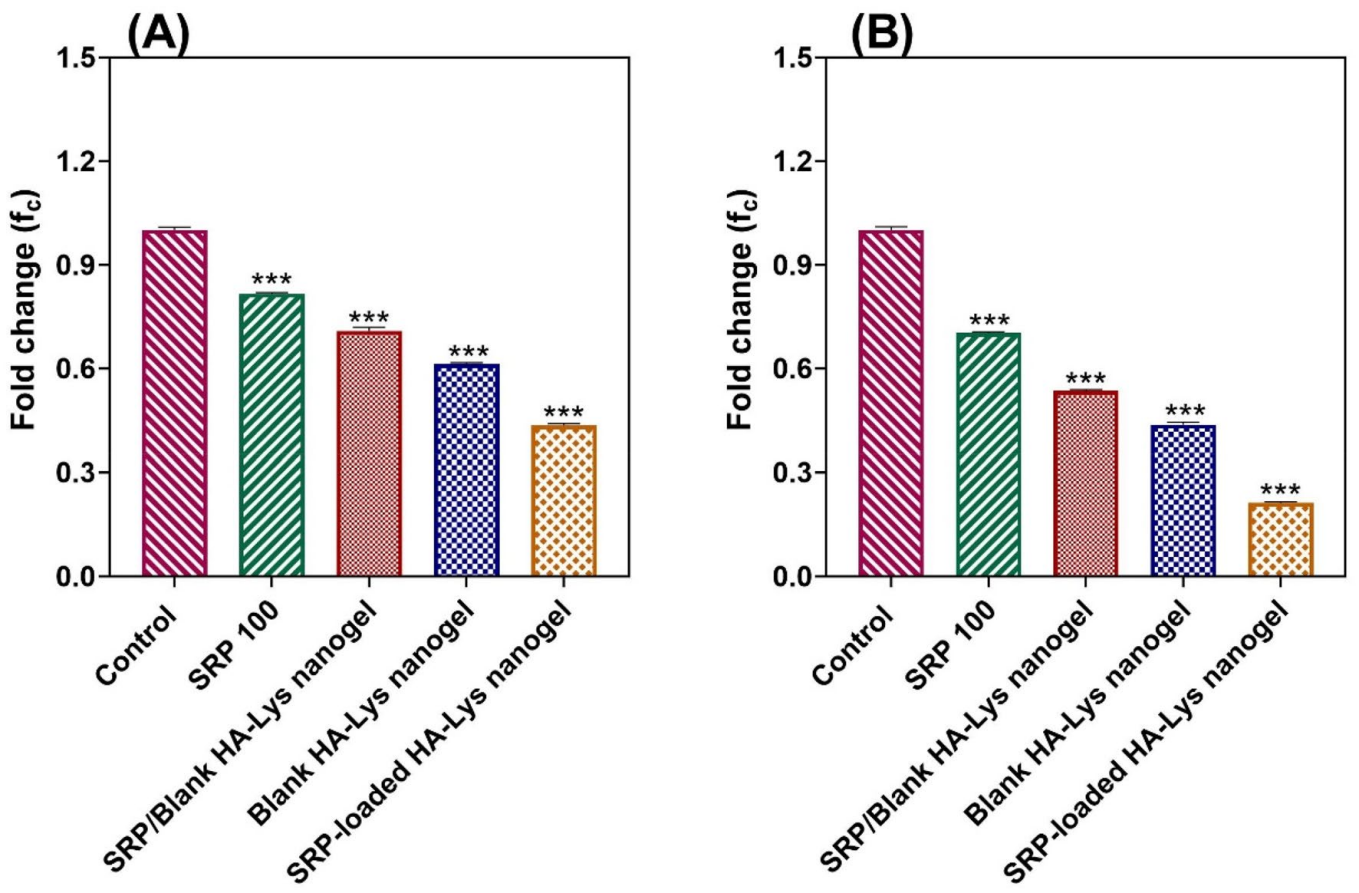
The fold changes of (**A**) *icaA* in *S. aureus* and (**B**) *ndvB* in *P. aeruginosa*; (Data are represented as mean ± SD; *** *p-value* < 0.001).

To gain more insights into the effectiveness of HA-Lys nanogel on the formation of *P. aeruginosa* and *S. aureous* biofilms, the expression level of specific genes was studied by real time PCR (Fig. 9A, B). SRP-loaded HA-Lys nanogel 100% treatment results in the highest reduction of genes expression and blank HA-Lys nanogel 100% was places in the second place. In all species tested, the PCR test revealed lower expression of the genes *ndvB* and *icaA*, which are associated with biofilm formation, following treatment with SRP100%, SRP/blank HA-Lys nanogel 50%, blank HA-Lys nanogel 100%, and SRP-loaded HA-Lys nanogel 100% in comparison to untreated samples (*p-value* < 0.001). These findings showed that HA-Lys nanogel can help SRP to get into bacterial cells and stop biofilms to be formed. The blank HA-Lys nanogel 100%, and SRP-loaded HA-Lys nanogel 100% might directly interact with certain transcription factors, leading to reduced *ndvB* and *icaA* expression.

## Discussion

There were opposite changes between size and scattered intensity attributed to the formation of complexes with strong aggregates and even precipitations. These results were consistent with other polyelectrolyte complexes consisting of polyelectrolytes with hydrophobic backbones^20^. According to the Sheetu Wadhwa study, since chitosan (CS) is cationic in nature : and HA is onionic it expected that a possibly close binding may retard the release of the loaded drug at the site of action and prolong the residence time of the carrier. These polysaccharides are expected to interact with each other, possibly through hydrogen bonding, ionic interactions, and other intermolecular forces. Therefore, different ratios of internal polymer including CS:HA 1:0.05–1:0.15 was further optimized concerning particle size, zeta potential, and entrapment efficiency^21^. Also, there was an increase in the numerical value of zeta potential after increasing the amount of drug concentration, while other independent variables were kept constant. An increase in HA leads to highly negative zeta potential because HA is an acidic polysaccharide with a negative charge. According to Fahmy et al. the incorporation of negatively charged HA causes extensive adsorption on the elastosomal^22^. However, the results showed that zeta potential increases linearly with enhancing stirring speed. This might be due to the agglomeration of individual particles that tends to reduce surface area and change the zeta potential value associated with that surface^23^. Increased PDI by raising the ratio of HA/Lys and drug concentration showed that an increase in these two factors may decrease nanogel stability^24,25^. But PDI decreased with increasing stirring speed. Moreover, the viscosity of the solution was reduced by elevating the stirring speed because it always exerts a constant amount of shear stress on the emulsifying mixture. As a result, the PDI of nanogels reduces due to the decrementof solution viscosity^23^. The average size of the optimized formulation is less than 100 nm. The particle diameter obtained by the DLS method is larger than SEM, which is attributed to the hydration effect of the SRP-loaded HA-Lys nanogel in aqueous medium^26^. The release profiles of SRP from HA nanogels were biphasic release processes^27,28^. The initial phase involves a relatively rapid release of the drug and is then followed by a slower release phase. The rapid drug release in the initial phase may be due to the desorption of the drug from the outer surface of the nanoparticles and slower drug release is mainly related to the diffusion of the drug through the nano structure^29–31^. A wide size distribution and heterogeneous particle sizes are observed as a result of temperature increment. The loaded protein content is another influencing factor that should be considered. The best sample, as can be observed, lost SRP in lesser extent when stored at 4 °C in comparison to 25 °C. Aggregation of the vesicles may induce a rise in their size as storage time increases. According to thermodynamic theory, surface energy is a size-dependent quantity. Smaller nanoparticles have a greater surface energy and tend to combine to reduce surface energy. The stability study showed that the size changes at 25 °C were significantly higher, as well as PDI and EE. Overall, nanoparticles seem to be more stable at 4 °C. Reduction in the activity of SRP after loading in nanogels can be attributed to electrostatic attachment of SRP with HA or the steric hindrance, size distribution of nanogels, and diffusion effect^32,33^ of SRP in nanogels. Despite the decreasing activity of SRP-loaded nanogels, according to previous reports, enzyme-loaded nanogels had better stability than free enzyme exposing environmental conditions. The results show that the loading process increases protease resistance against environmental fluctuations and improves enzymatic activity in the pharmaceutical industry and medicine because SRP loaded in HA nanogels can accumulate on the surface of bacterial cell walls^34^. Generally, blank HA-Lys nanogel showed significant microbial activity against *P. aeruginosa* and *S. aureus* compared to free enzyme. A possible explanation is because of the high antimicrobial properties of HA^35,36^. The antibacterial effect of HA itself have been reported before. For example Wenzhen et al. used HA as antibacterial agent to modify surface of microneedles^37^. Another reason may be due to poor penetration of free SRP in the biofilm compared with SRP-loaded HA-Lys nanogels. Also, the investigation of the samples’ effect on bacterial viability indicated that the reduction of bacterial viability in SRP-loaded HA-Lys nanogel was not significantly greater than blank HA-Lys nanogel. One of the primary mechanisms of SRPs therapeutic action is most likely its anti-biofilm activity. The anti-biofilm properties of SRP has been documented in an increasing number of research^38^. According to a prior study, SRP considerably boosted the efficiency of antibiotics against bacteria that produce biofilms^8^. In a comparable way, SRP was reported by Panagariya et al^39^. to augment the activity of other antibiotics, including ampicillin, cephalexin, diclacillin, minocycline, and cefotiam. In another investigation, Mecikogu et al. showed that treatment using SRP dramatically increased the action of antibiotics against bacteria that produce biofilms in animal models. According to Papa et al^7^. research, down regulation of surface proteins expression in pathogens that help biofilm formationis probably the way how the SRPs anti-biofilm function is mediated.

Similarly, some research has shown that SRPs activity is increased when it is incorporated into nanostructures. For example, Kumar et al^40^. discovered that the anti-inflammatory activity of SRP was increased by immobilizing the enzyme to magnetic nanoparticles utilizing glutaraldehyde as a chemical linker. In a different investigation, Kaur et al^41^. showed that arthritic inflammation was considerably reduced when SRP was conjugated to albumin nanoparticles via glutaraldehyde linkages.

## Conclusion

The present study focused on the preparation, optimization, and antibiofilm activity evaluation of SRP-loaded HA-Lys nanogels. SRP-loaded HA-Lys nanogel was prepared via the dropping method. The results of the Box-Behnken experimental design showed that polymer ratio, drug concentration, and stirrer speed had a converse effect on the particle size of blank HA-Lys nanogel. Nonetheless, the entrapment efficiency of nanogels is highly dependent on the quantity of enzyme. For the ideal sample, the average particle size was 156 nm, and the zeta potential was -14.1 mV. Moreover, the release of SRP reached about 77% after 72 h. Also, the physical characterization of blank HA-Lys nanogel indicated that nanogels had higher stability and lower changes in EE after 90 days at 4 °C compared to 25 °C.Anti-biofilm activity assessment showed the noticeable anti-biofilm effect of SRP-loaded HA-Lys nanogel against *P. aeruginosa* and *S.aureus*. Therefore, this nanocarrier can be considered as a suitable candidate for managing infections caused by biofilm forming pathogens.

## Data Availability

The datasets generated during and/or analyzed during the current study are available from the corresponding author upon a reasonable request.

## Abbreviations

HA: Hyaluronic acid
Lys: Lysine
SRP-loaded HA-Lys nanogel: Nanogels containing Serratiopeptidase
Blank HA-Lys nanogel: HA-Lys nanogel
BBD: Box-Behnken experimental design
SRP: Serratiopeptidase
*S. aureus*: *Staphylococcus aureus*
*P. aeruginosa*: *Pseudomonas aeruginosa*
PDI: Polydispersity index
EE%: Encapsulation efficiency percentage
GAG: Glycosaminoglycan
FT-IR: Fourier-transform infrared spectroscopy
